# Impact of a tobacco sales ban on the frequency of tobacco consumption in India during the COVID-19 pandemic

**DOI:** 10.18332/tid/161855

**Published:** 2023-04-28

**Authors:** Nitika Sharma, Mansi Chopra, Linda Bauld, Gaurang P. Nazar, Nishigandha Joshi, Aastha Chugh, Sailesh Mohan, Deepa Mohan, Mohammed K. Ali, Vishwanathan Mohan, Nikhil Tandon, K. M. Venkat Narayan, K. Srinath Reddy, Dorairaj Prabhakaran, Monika Arora

**Affiliations:** 1 Health Related Information Dissemination Amongst Youth, New Delhi, India; 2 Usher Institute, University of Edinburgh, Edinburgh, United Kingdom; 3 SPECTRUM Consortium, University of Edinburgh, Edinburgh, United Kingdom; 4 Public Health Foundation of India, Gurugram, India; 5 Centre for Chronic Disease Control, New Delhi, India; 6 Madras Diabetes Research Foundation, Chennai, India; 7 Rollins School of Public Health, Emory University, Atlanta, United States; 8 Emory Global Diabetes Research Center, Emory University, Atlanta, United States; 9 All India Institute of Medical Sciences, New Delhi, India

**Keywords:** tobacco, COVID-19, frequency, India

## Abstract

**INTRODUCTION:**

Measures to address the COVID-19 pandemic in India included a ban on the sale and use of tobacco products during 2020 when stay at home guidance (lockdown) was in place. In this study we examined the extent of reduction in frequency of tobacco consumption across all products.

**METHODS:**

Telephone survey was conducted between July and August 2020 across an existing cohort of tobacco users (n=801) residing in Delhi (55.4%) and Chennai (44.6%), India. The participants were recruited irrespective of their gender and use of any kind of tobacco product(s). The survey questionnaire was based on the STOP (Studying Tobacco users Of Pakistan) survey and adapted to the context of smoking and smokeless tobacco use in India.

**RESULTS:**

Cigarette consumption declined from a median value of 5.0 (IQR: 2–10) sticks in the pre-lockdown period to 2.0 (IQR: 0.4–5) sticks during the lockdown period. Reductions were reported in the daily use of bidis, from 8 (IQR: 4–12) sticks to 5 (IQR: 2–10) sticks and for smokeless tobacco users from 3.5 (IQR: 2–5) packs to 2 (IQR: 1–4) packs during the lockdown. Furthermore, the number of daily cigarette smokers in our cohort decreased from 32.6% (n=261) in the pre-lockdown period to 27.5% (n=220) during lockdown and smokeless tobacco users decreased from 35.8% (n=287) in pre-lockdown period to 30.3% (n=243) during the lockdown period.

**CONCLUSIONS:**

The decrease in tobacco use can be attributed to various societal and environmental factors. However, the pandemic-linked lockdown provided an opportune condition to reduce the use of tobacco products, which could be due to restricted access and increase in health awareness during the COVID-19 lockdown.

## INTRODUCTION

When the COVID-19 pandemic began, public health authorities around the world, including in India, targeted interventions on ways to reduce infection and COVID-19 associated morbidity and mortality^1^. This included advising people to stay at home and discouraging tobacco use, including bans in public places, and health education. Public health measures against tobacco use during the COVID-19 pandemic included information that SARS-CoV-2 can spread through droplets of saliva discharged from the nose or mouth^1^. Smokeless tobacco users engage in spitting behaviors, an activity that could pose an infection risk.^1^ In addition, even early in the pandemic there was concern that smokers may be at increased risk of COVID-19 disease severity because of the links between respiratory illness and smoking, and the fact that COVID-19 is (at least in part) a respiratory disease^2^. People with tobacco attributable co-morbidities like cardiac and pulmonary disorders, cancers, diabetes and other non-communicable diseases have a poor prognosis with COVID-19^3^.

**Figure 1:**
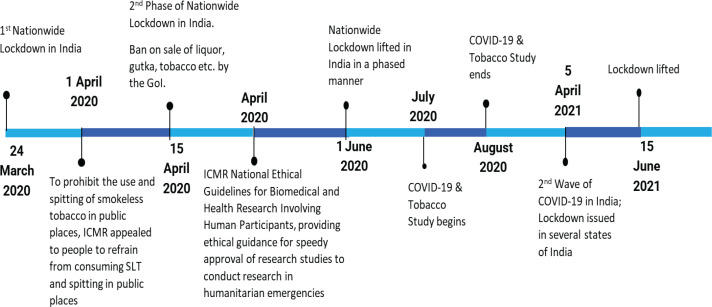
Timeline of the study with respect to COVID-19 national lockdown and tobacco sales ban in India

In India, under the Disaster Management Act 2005, 28 states and Union territories included the use of punitive provisions to ban the use of tobacco products and spitting in public places, which were enforced from April 2020 to June 2020^4^. Additionally, the Indian Council of Medical Research (ICMR)^5^ also advised tobacco users about the potential infection risks of spitting and the link between COVID-19 disease severity and smoking (Figure 1).

A previous study in India conducted on tobacco users enrolled in a cessation program, suggested that limited access when COVID-19 protective measures were in place including stay at home guidance (‘lockdown’), contributed to them stopping tobacco use. However, the study comprised tobacco users already motivated to quit tobacco and did not examine the changes in tobacco use frequency among users^6^. Our earlier published study suggested that tobacco users overall made two quit attempts during the COVID-19 lockdown in 2020. We identified that the reduced availability of tobacco products in India during the first lockdown seems to have provided a conducive environment for tobacco users to quit this addiction^7^. With that background, we examined data from the same study to assess the effect of the sales ban during lockdown on the frequency of smoked (cigarettes, bidis) and smokeless tobacco use among tobacco users as well as any differences based on sociodemographic characteristics.

## METHODS

### Study design, setting and participants

A sample of tobacco users participating in the ongoing CARRS (**C**entre for c**A**rdiometabolic **R**isk **R**eduction in **S**outh Asia) longitudinal study were surveyed for changes in tobacco use during the pandemic. The CARRS cohort consists of representative samples in two urban cities, Delhi and Chennai, and the detailed methodology is published elsewhere^7,8^.

This cross-sectional study was conducted July and August 2020. The survey questionnaire was based on the STOP (Studying Tobacco users Of Pakistan)^9^ survey and adapted to the context of smoking and smokeless tobacco use in India. The questionnaire was translated into English and regional languages, Hindi (for Delhi participants) and Tamil (for Chennai participants)^7^. At the time of the survey, the participants were asked about the frequency of their tobacco use (cigarettes, bidis, and smokeless tobacco like zarda, khaini, paan masala etc.) before and during the COVID-19 lockdown in the survey^7^.

### Participant recruitment and data collection

Participants aged >20 years, irrespective of their gender, using any form of tobacco, well versed with the questionnaire languages and those who agreed to provide consent were enrolled in the study (with a response rate 60.8%)^7^. Tobacco users (n=253) who had quit using tobacco (more than 3 months before the date of the survey, i.e. after the lockdown) were excluded from the study. Additionally, participants suffering from any severe illness, institutionalized, unable to respond to the survey and not willing to provide or record verbal consent (n=138) were also excluded from the study.

The objectives of the study were explained to the study participants and only after seeking their audio recorded verbal informed consent, a telephone interview using a structured questionnaire was conducted. Prior ethics approval for research involving human subjects for this study was obtained from the Institutional Ethics Committee of the Centre for Chronic Disease Control.

### Data analysis

The frequency variable (outcome) was reported as the number of sticks (per day/per week/per month) (for cigarettes and bidis) and number of packets (per day/per week/per month) (for smokeless tobacco) and was treated as a continuous variable in the analysis. The users reporting tobacco use every day were labelled as daily tobacco users, while those who reported using tobacco weekly or monthly were labelled as occasional tobacco users. For analysis, weekly frequency was converted into daily frequency by dividing by 7. Since the outcome variable was skewed even after log transformation, bivariate associations were studied using non-parametric tests such as Mann Whitney U test/Wilcoxon signed rank test and Kruskal Wallis test as appropriate. STATA 13.0 (StataCorp, LP, Texas) was used for conducting analyses.

## RESULTS

Overall, 801 participants were enrolled from Delhi (55.4%) and Chennai (44.6%). The response rate calculated using the CASRO estimator was 60.9%^7^. Of the total participants, 38.1% were cigarette smokers (n=305), 24.3% were bidi smokers (n=195), and 40.4% were SLT users (n=324). A significant reduction in the frequency of daily tobacco consumption (cigarettes, bidis, and smokeless) was reported among all daily tobacco users (p<0.05) (Table 1). However, in the case of occasional tobacco users, the decline in weekly frequency was only significant for bidis and smokeless tobacco. The daily reported number of cigarette sticks for cigarette smokers significantly declined from a median of 5 sticks (IQR: 2.0–10.0) in the pre-lockdown period to 2 sticks (IQR: 0.4–5.0) during the lockdown period (Table 1). Similarly, significant reductions were reported in daily use of number of sticks of bidis before the lockdown period from 8 (IQR: 4–12) to 5 (IQR: 5–10) during the lockdown period, and number of packs of smokeless tobacco before lockdown of 3.5 (IQR: 2–5) to 2 (IQR: 1–4) during the lockdown period. In bivariate analyses, significant reductions in daily consumption were reported among cigarette and smokeless tobacco users in both cities (p<0.05). There were no statistically significant associations between reductions in consumption and the socioeconomic characteristics of participants (Table 1).

**Table 1:** Change in frequency of daily use of tobacco products from before to during the COVID-19 lockdown with sociodemographic variables

	*Cigarettes (sticks) Median (IQR)*		*p*	*Bidis (sticks) Median (IQR)*		*p*	*Smokeless tobacco (packs) Median (IQR)*		*p*
	*Before*	*During*		*Before*	*During*		*Before*	*During*	
**Daily users** (daily frequency)	5 (3–10)	4 (2–7)	**0.00^c^**	8 (4–12)	6 (3–10)	**0.00^c^**	4 (2–5)	3 (1–5)	**0.000^c^**
**Occasional users** (weekly frequency)	1 (0.5–2)	2 (0.5–2)	0.56	2 (1–3)	1 (1–2)	**0.00**	2 (1–3)	1 (1–2)	0.05^c^
**Overall daily frequency^a^** (for both daily + occasional users)	5 (2–10)	2 (0.4–5)	**0.000^c^**	8 (4–12)	5 (2–10)	**0.000^c^**	3.5 (2–5)	2 (1–4)	**0.000^c^**
**Gender**			0.91			0.89			0.20
Male	5 (2–10)	2 (0.4– 5)		8 (4–12)	5 (2–10)		4 (2–5)	2 (1– 4)	
Female	2 (1–6)	2 (0–2)		5.5 (4–12)	5 (4–12)		3 (2–5)	3 (0.42– 4)	
**City**			**0.00^d^**			0.66			**0.00^d^**
Delhi	3 (1–6)	2 (0.3–5)		7 (4–12)	6 (2–10)		4 (2–5)	3 (1– 5)	
Chennai	5 (3–10)	3 (0.9–5)		10 (3–20)	5 (2–10)		3 (1–5)	2 (0.28– 3)	
**Age** (years)^b^			0.95			0.41			0.07
25–44	4 (1–8)	2 (0.1–4)		7.5 (3–12)	5 (1.5–10)		3 (2–5)	2 (0.29– 4)	
45–64	5 (3–10)	3 (1–5)		7 (4–12)	6 (2–10)		4 (2–5)	2 (1– 4)	
≥65	6 (2–15)	4 (2–10)		8 (5–12)	5 (4–10)		4 (3–5)	3 (2– 4)	
**Education level^b^**			0.85			0.58			0.93
Illiterate	5 (1–10)	2 (0.43–10)		7 (4–12)	6 (3–12)		4 (3–6)	3 (1– 5)	
Professional degree/postgraduate	6 (2–6)	2 (1–6)		20 (20–20)	4 (4–4)		1.5 (0.64–2.5)	0.57 (0.07–2)	
Graduate (BA/BSc/BCom/Diploma)	5 (2–7)	2 (0.29–5)		5 (4–8)	2 (2–6)		3 (2–5)	2 (1– 4)	
Secondary school/Intermediary	5 (2–10)	3 (1–5)		7.5 (4–12)	5 (2–10)		4 (2–5)	2 (0.29– 5)	
High school (classes 5 to 9)	4 (2–10)	2 (0.14–5)		7 (4–12)	5 (2–10)		3 (2–5)	2 (1– 4)	
Primary school (up to class 4)	6 (5–10)	4 (1.5–5)		10 (6.5–18)	10 (4.5–13.5)		4 (2–5)	2.5 (1 – 5)	
**Employment status^b^**			0.75			0.71			0.38
Employed	5 (2–10)	2 (0.43–5)		8 (4–12)	6 (2–10)		3 (2–5)	2 (0.43– 4)	
Student	5 (2–6)	2 (1–5)		5 (3–7)	4.5 (3–8)		4 (2–5)	3 (1– 5)	
Housewife	1 (1–1)	-		4.5 (4–8.5)	3 (2–8)		3 (1–4)	2.5 (1 – 3)	
Retired	10 (10–20)	8 (4–10)		9 (7–15)	6.5 (2.5–9)		4 (3–4)	3 (2– 4)	
Unemployed	15 (10–20)	10 (5–12)		12.5 (10–20)	9.5 (5–15)		3 (2–4)	3 (2– 3)	

a For overall daily frequency the occasional tobacco users weekly frequency was converted to daily frequency.

b p value estimates using Kruskal Wallis H test.

c p value<0.05 estimated using Wilcoxon matched pair signed rank test.

d p value<0.05 estimated using Mann Whitney/Wilcoxon rank sum test. IQR: interquartile range.

The number of daily cigarette users decreased during the COVID-19 national lockdown period (before lockdown, 261 vs during lockdown, 220), and the number of occasional users for cigarettes increased during the lockdown period (before lockdown, 41 vs during lockdown, 51). Similarly, the number of daily bidi users (before lockdown, 189 vs during lockdown, 185) as well smokeless tobacco (before lockdown, 287 vs during lockdown, 243) also decreased during the lockdown period. While there was no change in numbers among occasional users for bidis, there was a slight decrease in the number of occasional users for smokeless tobacco during the lockdown period (before lockdown, 36 vs during lockdown, 34).

## DISCUSSION

The findings of this study provide preliminary insights on the change in frequency of smoking (both cigarettes and bidis) and smokeless tobacco use in 2020 during a lockdown period to address COVID-19. People were advised to stay at home, tobacco sales were banned and public information campaigns about potential links between tobacco use and COVID-19 infection and disease severity were implemented. We observed a significant reduction in the frequency of all forms of tobacco use (cigarettes, bidis, and smokeless tobacco) among tobacco users. In addition, the number of daily tobacco users of cigarettes, bidis and smokeless tobacco decreased during the lockdown period compared to the pre-lockdown period. This is in line with our previous study conducted in India which found evidence of cessation across all smoked and smokeless tobacco products due to restricted access and increased knowledge among current users on the harmful effects of tobacco use and COVID-19^7^. In contrast, a study based on Google trends on the Indian population indicated that the lockdown did not increase the quitting intention among the smokers^10^. However, the study was based on search history of Google trends during the start of the pandemic when people were more inclined towards other preventive measures rather than prioritizing to quit smoking. Another study from Pakistan during the same period had also reported significant bidirectional changes in smoking patterns since COVID-19, with the majority of participants reporting a decrease in frequency of smoking^11^. In contrast, a few studies from other countries found no change or even an increase in tobacco use during the pandemic^12,13^. This might be due to different policies introduced during lockdown periods compared to India.

The effectiveness of tobacco sales bans is dependent on the comprehensiveness of the legislation, level of enforcement and public support for a ban^14^. The combination of a sales ban and health information during the 2020 lockdown in India might have contributed to reducing frequency of consumption among daily tobacco users. Additionally, the opportunity to access tobacco products only at the time of shopping essential products (further subject to their availability in the stores) might have also limited the access to tobacco products, thereby resulting in reduction of tobacco use frequency.

### Limitations

Limitations of the study include that the research was only conducted in two cities in India and excluded tobacco users in other urban and rural centers. In addition, the survey relied on self-report measures and there was no biochemical validation of tobacco use status or reduction in consumption. Furthermore, our study design can only provide indications and cannot attribute causality. Overall in our results, no significant bivariate associations were reported with other independent variables. Therefore, multivariate regression analysis was not undertaken.

## CONCLUSIONS

The reduction in tobacco use can be attributed to various societal and environmental factors. However, the pandemic-linked lockdown provided an opportune condition to reduce the use of tobacco products, which could be due to restricted access and increase in health awareness during the COVID-19 lockdown.

## Data Availability

The data supporting this research are available from the authors on reasonable request.
