# Corrigendum: Impact of a tobacco sales ban on the frequency of tobacco consumption in India during the COVID-19 pandemic

**DOI:** 10.18332/tid/166113

**Published:** 2023-06-08

**Authors:** Nitika Sharma, Mansi Chopra, Linda Bauld, Gaurang P. Nazar, Nishigandha Joshi, Aastha Chugh, Sailesh Mohan, Deepa Mohan, Mohammed K. Ali, Vishwanathan Mohan, Nikhil Tandon, K. M. Venkat Narayan, K. Srinath Reddy, Dorairaj Prabhakaran, Monika Arora

**Affiliations:** 1 Health Related Information Dissemination Amongst Youth, New Delhi, India; 2 Usher Institute, University of Edinburgh, Edinburgh, United Kingdom; 3 SPECTRUM Consortium, University of Edinburgh, Edinburgh, United Kingdom; 4 Public Health Foundation of India, Gurugram, India; 5 Centre for Chronic Disease Control, New Delhi, India; 6 Madras Diabetes Research Foundation, Chennai, India; 7 Rollins School of Public Health, Emory University, Atlanta, United States; 8 Emory Global Diabetes Research Center, Emory University, Atlanta, United States; 9 All India Institute of Medical Sciences, New Delhi, India

**Keywords:** tobacco, COVID-19, frequency, India

Corrigendum on:

Impact of a tobacco sales ban on the frequency of tobacco consumption in India during the COVID-19 pandemic

By Nitika Sharma, Mansi Chopra, Linda Bauld, Gaurang P. Nazar, Nishigandha Joshi, Aastha Chugh, Sailesh Mohan, Deepa Mohan, Mohammed K. Ali, Vishwanathan Mohan, Nikhil Tandon, K. M. Venkat Narayan, K. Srinath Reddy, Dorairaj Prabhakaran, Monika Arora

Tobacco Induced Diseases, Volume 21, Issue April, Pages 1–6, Publish date: 28 April 2023

DOI: https://doi.org/10.18332/tid/161855

In the originally published version of the article, the name of the 12th author was given as Venkat K. M. Narayan and is now changed to K. M. Venkat Narayan. Furthermore, in the authors’ contributions section, the initials VKMN are now changed to KMVN. The mentioned changes are corrected also online.

